# The impact of Baby Friendly Initiative accreditation: An overview of systematic reviews

**DOI:** 10.1111/mcn.13216

**Published:** 2021-06-01

**Authors:** Frankie J. Fair, Alison Morrison, Hora Soltani

**Affiliations:** ^1^ College of Health, Wellbeing and Life Sciences Sheffield Hallam University Sheffield UK

**Keywords:** baby friendly initiative, breast feeding, breast milk, overview of reviews

## Abstract

Despite its reported benefits, breastfeeding rates are low globally, and support systems such as the Baby Friendly Initiative (BFI) have been established to support healthy infant feeding practices and infant bonding. Increasingly reviews are being undertaken to assess the overall impact of BFI accreditation. A systematic synthesis of current reviews has therefore been carried out to examine the state of literature on the effects of BFI accreditation. A systematic search of CINAHL, MEDLINE, Maternal and Infant Health, Scopus, the Cochrane Library and PROSPERO was undertaken. Study selection, data extraction and critical appraisal of included reviews using the AMSTAR‐2 tool were undertaken by two authors, with disagreements resolved through discussion with the third author. Due to heterogeneity, a narrative synthesis of findings was applied. Fourteen reviews met the inclusion criteria. Overall confidence in the results of the review was rated as high for three reviews, low for two reviews and critically low for nine reviews. Most evidence suggests some increase in breastfeeding initiation, exclusivity and duration of breastfeeding, and one main trial suggests decreased gastrointestinal infection and allergic dermatitis in infants. However, overall certainty in the evidence was rated as very low across all outcomes due to concerns over risk of bias within and heterogeneity between the original studies. More contemporary, good‐quality randomised controlled trials or well‐controlled prospective comparative cohorts are required to better evaluate the impact of full BFI accreditation, with particular attention paid to the context of the research and to long‐term maternal and infant health outcomes.

**Key messages**

- Current evidence suggests there may be some improvement in initiation and breastfeeding duration with Baby Friendly Initiative accreditation, especially in low‐income countries; however, confidence in these findings was very low. There is minimal evidence of the impact of BFI accreditation on maternal and infant health outcomes.
- The majority of current evidence assessing BFI accreditation was of poor methodology or at high risk of bias.
- More contemporary, good‐quality randomised controlled trials or well‐controlled prospective comparative cohorts are required to better evaluate the impact of BFI accreditation.
- Particular attention is needed to the context of the research, both background socio‐economic and breastfeeding practices, and to explore longer term outcomes to see if benefits in breastfeeding duration are sustained.

## 1 INTRODUCTION

The World Health Organization (WHO) recommends that breastfeeding is initiated within 1 h of birth (United Nations Children's Fund, 2016) and that infants are exclusively breastfed until 6 months of age, with continued breastfeeding alongside introduction of solid foods thereafter (World Health Organisation, 2001). A recent systematic review of randomised and quasi‐randomised trials suggested that there is no evidence to move away from the guidance to exclusively breastfeed infants for the first 6 months of life (Smith & Becker, 2016). Evidence suggests that infants fed with breastmilk substitutes are at increased risk of gastrointestinal infections, respiratory infections, asthma, coeliac disease and sudden infant death as well as increased risk of obesity and diabetes in later life (Lessen & Kavanagh, 2015; Victora et al., 2016). Numerous adverse outcomes are also increased in mothers who do not breastfeed their infants, including ovarian cancer, breast cancer, type 2 diabetes and postnatal depression (Gunderson et al., 2018; Lessen & Kavanagh, 2015; Victora et al., 2016). Worldwide, an estimated 823,000 deaths in children under five and up to 20,000 deaths a year from breast cancer could be prevented by improving breastfeeding practices (Victora et al., 2016). Although breastfeeding can protect against child and maternal deaths in low‐, middle‐ and high‐income countries, disparities in the magnitude of different health benefits according to country income level are known to exist (Victora et al., 2016).

Global data show the prevalence of breastfeeding at 6 and 12 months decreases with increasing national wealth, with prevalence of breastfeeding at 12 months decreasing 10% for each doubling in gross domestic product per head (Victora et al., 2016). As well as national wealth, breastfeeding rates are lower in women who are younger, of low socio‐economic status, living in deprived areas, of lower educational attainment, who smoke (Cohen et al., 2018; McAndrew et al., 2012) and in women with a raised body mass index (Wojcicki, 2011).

Despite its reported benefits, breastfeeding rates up to 6 months or longer appear to be low globally (Victora et al., 2016), and systems such as the UNCIEF Baby Friendly Initiative have been established to support healthy infant feeding practices. The Baby Friendly Hospital Initiative (BFHI) was developed in 1991 and updated in 2018, with the aim for every baby to have the best start in life through the global protection, promotion and support of breastfeeding in facilities providing maternal and newborn services (United Nations Children's Fund & World Health Organization, 2018). Each facility is required to comply with the 10 steps to successful breastfeeding, which incorporate adherence to the WHO Code for Marketing of Breastmilk Substitutes, policy development, staff training and key clinical practices for supporting breastfeeding (see Appendix A for full details of these steps). The BFHI initiative has also been expanded to include a 7‐point plan for community services to support sustained breastfeeding (UNICEF, 2014). To become accredited, community facilities are required to have a written breastfeeding policy, training for staff, and provide a supportive and welcoming atmosphere for breastfeeding women and work collaboratively with the aim for increased exclusivity and duration of breastfeeding (see Appendix B). Each country adopts the Baby Friendly Initiative into its own framework for accreditation (for example BFHI Australia, 2020; UNICEF UK, 2017). Of the 155 countries included within a WHO survey, 71% had an operational BFHI programme in 2016–2017; however, only six countries reported that the majority of their facilities had BFHI accreditation (WHO, 2017). Overall, coverage was estimated to be 10%, although there were wide variations between and within regions—for example, in Europe over half of births occurred within BFHI accredited facilities within 13 countries, but 12 different European countries had no accredited facilities (WHO, 2017).

Many systematic reviews have evaluated evidence behind individual steps of the Baby Friendly Initiative (Jaafar, Ho, Jahanfar, & Angolkar, 2016; Jaafar, Ho, & Lee, 2016; Lumbiganon et al., 2016; Moore et al., 2016; Smith & Becker, 2016). Increasingly reviews have also been focusing on the assessment of the overall impact of BFI accreditation. As systematic reviews are increasingly published, clinicians can be left feeling overwhelmed by the plethora of evidence. Therefore, the requirement for overviews of reviews is gaining recognition to enable systematic reviews to be compared and the evidence collated to provide an overall understanding of the available information on a given topic (Aromataris et al., 2014). The aim of this overview was therefore to evaluate the quality and extent of systematic evidence regarding the impact of Baby Friendly Initiative accreditation in order to better understand the effectiveness of this global intervention on breastfeeding rates and health related outcomes. Consideration was given to the income level of the country of the original trials and the level of BFI accreditation when evaluating the evidence.

## 2 METHODS

### 2.1 Search strategy

The review was undertaken in accordance with the pre‐planned protocol (PROSPERO CRD42020171859). A systematic search of CINAHL, MEDLINE, Maternal and Infant Health, Scopus, the Cochrane Library and PROSPERO registry of systematic reviews was undertaken. The WHO website was also searched for relevant publications. The search strategy included terms around ‘baby friendly initiative’ and ‘systematic review’. An example full search strategy within one database can be found in Appendix C. Databases were searched from 1991 when the Baby Friendly Initiative was launched to 6 March 2020. Systematic reviews were limited to those published in the English language. Reference lists of included systematic reviews and other relevant literature were screened manually for further citations.

### 2.2 Inclusion and exclusion criteria

Retrieved citations were screened by two independent researchers against the eligibility criteria (Table 1) by title and abstract and then full text for relevant articles; any disagreements were resolved through discussion with the third author. Authors of citations of conference proceedings or protocol registrations were contacted to enquire after full text articles.

**TABLE 1:** Eligibility criteria

Inclusion criteria	Exclusion criteria
Systematic reviews that evaluated Baby Friendly Initiative accreditation through any type of research including randomised controlled trial, controlled trials, cross‐sectional and cohort studies OR Systematic reviews looking at any breastfeeding interventions, provided that separate subgroup analysis is provided for Baby Friendly Initiative accreditation	Systematic reviews focusing just on individual steps of BFI accreditation, rather than BFI implementation and accreditation overall
Articles were in English language and full text articles could be obtained	Any form of review without an explicit search strategy
Included studies within the systematic review could look at pregnant, or recently postnatal women or at maternity units where full BFI accreditation was compared to either non or partial BFI accreditation	
Breastfeeding intention, initiation or duration were reported within the systematic review	
Included studies within the systematic reviews could be undertaken in any country, i.e., high‐, middle‐ or low‐income.	

### 2.3 Data extraction

Data extraction was undertaken by two researchers using a pre‐defined data extraction table. Authors of the systematic reviews were contacted where required for additional information.

### 2.4 Risk of bias assessment

Two reviewers independently assessed included systematic reviews for risk of bias using the Assessment of Multiple Systematic Reviews v2 (AMSTAR‐2) checklist (Shea et al., 2017). See Appendix D for the full checklist. Disagreements were resolved through discussion with the third reviewer. The checklist authors (Shea et al., 2017) believe seven domains within the checklist can critically affect the validity of the review and its conclusions but acknowledge that reviewers can add or substitute domains as required according to the nature of the systematic reviews appraised. Within this overview, nine AMSTAR‐2 domains were considered to be critical. Seven of these coincide with areas considered critical by the checklist authors (Shea et al., 2017), including Item 2: review methods established prior to conducting review; Item 4: comprehensive literature search; Item 7: justification for excluding individual studies; Item 9: satisfactory techniques for assessing risk of bias within included trials; Item 11: appropriate methods for statistically combining results; Item 13: risk of bias considered when interpreting/discussing review results and Item 15: assessment for presence of publication bias. Given disparities in breastfeeding outcomes between countries and the wide range of sociocultural determinants that can impact upon breastfeeding Item 14: heterogeneity of included studies discussed was also considered critical for this review. Item 12: the impact of risk of bias considered on meta‐analysis results was also considered a critical domain within this overview. An overall rating of confidence in the results was given depending on the presence of flaws in the above critical domains or other weaknesses identified within the systematic review in accordance with the criteria set out in Shea et al. (2017); high overall confidence where there was no or one weakness within a non‐critical domain, moderate overall confidence where there was more than one weakness in a non‐critical domain, low overall confidence where there was one critical weakness, with or without other weaknesses in non‐critical domains or critically low confidence in the results where there was more than one weakness in a critical domain. For the purposes of this review, no weakness was considered to have occurred within the domain if the criteria were fully or partially met.

### 2.5 Data synthesis

Using a narrative synthesis approach, a formal discussion of the results of the systematic review evidence base regarding the impact of Baby Friendly Initiative accreditation was undertaken. This included an assessment of whether the cumulative evidence suggested breastfeeding and other outcomes such as maternal and infant wellbeing were improved with Baby Friendly Initiative accreditation, or whether there was a lack of evidence and/or inconclusive results.

A ‘Summary of Findings’ table was produced using the GRADE approach (Schünemann et al., 2013) to indicate for each finding the quality of the systematic reviews reporting that outcome, as well as the quality of the original studies included within the review. An overall grade—high, moderate, low or very low—was assigned to each outcome to reflect confidence in the current evidence.

Subgroup analyses were planned to look at differences in outcomes according to income level of the country of original trials (high, middle and low) and according to stage of Baby Friendly Initiative accreditation.

## 3 RESULTS

Of the 316 citations identified after removing duplicates, 54 articles were screened at full text. Of these, 16 articles (covering 14 separate systematic reviews) were included (see flow diagram of study selection in Appendix E). Appendix F provides reasons for exclusion at full text.

### 3.1 Characteristics of included systematic reviews

Characteristics of the included systematic reviews can be found in Table 2. Thirteen systematic reviews looked at BFI within a hospital and/or community setting, with one examining breastfeeding promotion within the neonatal intensive care unit (NICU) setting (Renfrew et al., 2010). The included systematic reviews predominantly used a narrative synthesis approach as meta‐analyses was inappropriate due to the high degree of heterogeneity within included studies in relation to study design, intervention and definitions of outcomes (initiation, duration and exclusivity). Only four systematic reviews performed meta‐analyses (Chung et al., 2008; Kim et al., 2018; Sinha et al., 2015, 2017). BFI was the sole intervention in four systematic reviews (Atchan et al., 2013; Fallon et al., 2019; Munn et al., 2016; Pérez‐Escamilla et al., 2016) with the remainder exploring any interventions aimed at improving breastfeeding initiation, duration and exclusivity but included BFI as a subgroup. Two reviews exclusively included randomised controlled trial evidence or quasi‐randomised studies (Chung et al., 2008; Kim et al., 2018), one review included quantitative studies and systematic reviews (Feltner et al., 2018), three included research of any methodology (Beake et al., 2012; Fallon et al., 2019; Munn et al., 2016) and one review included quantitative, mixed methods or systematic review studies (Hannula et al., 2008), with the remaining reviews incorporating quantitative research that included randomised or observational study designs. The reviews included a total of 105 individual studies that were attributed by the reviews to be evaluating BFI implementation. The number of BFI implementation studies in each review ranged from 2 (Chung et al., 2008; Fairbank et al., 2000) to 58 (Pérez‐Escamilla et al., 2016). A detailed look at each study revealed 25 studies examined breastfeeding rates according to the number or type of BFI‐related practices received rather than BFI accreditation per se, 18 only looked at a specific component of BFI accreditation such as education or rooming‐in, 5 looked at structured organisational interventions that were not BFI and 6 were qualitative studies, with the remaining 51 quantitatively evaluating full BFI implementation. The vast majority of studies included within the systematic reviews were conducted in high (62%) and upper middle‐income (30%) countries (The World Bank, 2020), with only one study conducted in a low‐income country (Democratic Republic of Congo).

**TABLE 2:** Characteristics of included studies

Author	Review aim	Search strategy	Studies and participants Number of BFI intervention studies Country of origin of included studies	Population, Intervention, Comparator, Outcome (PICO)	Risk of bias tool	Author's conclusions
Atchan et al. (2013)	To present a synthesis of issues related to BF and BFHI and to discuss the uptake and support of BFHI using Australia as a case study	2 databases and reference lists searched English language only Searched 1991 to current	Total number of participants not recorded Total number of included studies not clearly defined. **14 BFHI intervention studies**—1 RCT, remainder observational studies Multiple countries—high and low‐income	*Population:* Women who give birth in a BFI or non‐BFI hospital, both in Australia and internationally *Intervention:* BFI accreditation *Comparator:* Not reported *Outcome:* BF outcomes in high‐income countries	No evidence of quality assessment	No causal link demonstrated between BFHI and increase duration and exclusivity of BF, however positive association is probable Clear outcome definitions required
Beake et al. (2011) and Beake et al. (2012)	To assess whether structured BF programmes such as BFI have higher initiation and exclusive BF duration rates	13 databases including trial registers, as well as reference lists searched Grey literature searched English language only Searched 1992 to 2010	In individual studies from 400 to 464,246 infants 26 studies in total: 5 systematic reviews, 1 RCT, 2 controlled trials, the remainder observational studies **For most studies BFHI was the ‘structured’ intervention** 15 studies high‐income countries, 6 upper middle‐income	*Population:* Pregnant women and mothers of newborn infants in hospital *Intervention(s):* ‘Structured programme’ to support BF, either multi‐faceted (organisational, service delivery, and individual behaviour level) e.g. BFHI, or single faceted e.g. locally developed staff education *Comparator:* No structured BF support in place *Outcomes:* Primary—BF initiation and duration	Joanna Briggs methodological quality checklist	Structured programmes may be of most benefit in settings with low initiation and duration rates
Chung et al. (2008)	To systematically review evidence for the effectiveness of primary care–initiated interventions to promote BF with respect to BF, child and maternal health outcomes	3 databases including trial register, as well as reference lists searched English language only Searched September 2001 to February 2008	29,020 participants (BFHI studies 17,396 participants) 38 studies in total: RCTs (including quasi‐randomised) **2 evaluated BFI implementation** High‐income countries except for the 2 BFHI studies, conducted in Brazil and Belarus	*Population:* Pregnant families and new families of term infants *Intervention:* System‐level BF support (e.g. BFHI or staff training), formal BF education, professional support or lay support *Comparator:* Any usual prenatal, peripartum, or postpartum care, as defined in each study *Outcomes:* BF initiation, duration, exclusivity	U.S. Preventive Services Task Force criteria	BF interventions increase short‐ and long‐term exclusive BF. Lay support and interventions delivered in both antenatal and postnatal periods most effective BFHI interventions increased exclusive BF up to 6 months
Fairbank et al. (2000)	To evaluate which promotion programmes are most effective at increasing the number of women initiating BF	15 databases including trial register, as well as reference lists searched. Hand searching of some sources Experts and lay groups contacted No country, language or date restrictions Searched inception to November 1998	Total number of participants not recorded 59 studies in total; RCTs and observational studies **2 specific BFHI studies**—1 RCT and 1 before‐after Range of income status of countries. BFHI specific studies—Brazil and Thailand	*Population:* Pregnant women and mothers of newborn infants in the immediate postpartum period. Women who may breastfeed in the future also included *Intervention:* Any type of intervention designed to promote initiation of BF *Comparator:* Standard or routine care, or an alternative BF promotion programme *Outcomes:* The rate of initiation of BF. Secondary outcome—duration and/or exclusivity of BF	“Comprehensive methodological checklist”	Institutional change interventions whether part of BFI or independent were effective at increasing initiation and duration of BF, especially in low‐income countries
Fallon et al. (2019)	1. Examine the impact of BFI on maternal and infant health outcomes in the UK 2. examine women's views and experiences of BFI ‐compliant care in the UK	3 databases and reference lists searched Included only published literature of UK studies Searched 1991‐August 2017	141 to 464,266 in the quantitative studies; 15 to 72 in qualitative studies **11 studies in total all BFI**: 6 observational studies, 5 qualitative studies UK only	*Population:* Pregnant women, postnatal women, infants *Intervention:* Baby friendly initiative *Comparator:* N/A *Outcome:* Maternal mental health (depression, anxiety, quality of life), infant morbidity and mortality, BF outcomes	CASP cohort and qualitative checklists and adapted intervention and cross‐sectional CASP checklists	Future work is required to assess BFI effectiveness on longer term maternal and infant health outcomes in the UK Current BFI delivery does not meet women's needs and sets unrealistic expectations of BF
Feltner et al. (2018)	To summarise the effectiveness of community, workplace and health‐system based programs and policies to support BF and association between BF and maternal health	4 databases including trial register, as well as reference lists searched English language only Searched 1980 to October 2017	1,244,228 women included in BFHI studies 40 studies in total looked at policies or programs **12 studies on BFI**—including 1 RCT, remainder observational For BFI studies: 9 conducted in high‐income countries, 3 in upper middle‐income countries	*Population:* Childbearing women *Intervention:* Community, workplace and healthcare system‐based interventions to promote and support BF including BFHI *Comparator:* Concurrent control group or including multiple pre‐ and post‐measures of BF rates *Outcomes:* BF initiation, duration and exclusivity	Cochrane risk of bias for trials and ROBIS‐I for observational studies and predefined criteria from AHRQ methods guide	BFI is associated with improved rates of BF initiation and duration
Hannula et al. (2008)	To explore the effectiveness of interventions at supporting BF	3 databases including trial register searched Articles in English, Finnish or Swedish Searched 2000 to March 2006	Studies varied from <50 to >1,000 31 studies in total plus 5 reviews Quantitative and mixed‐methods **6 studies were BFI focussed** For the 6 BFI studies: 5 high‐income and 1 upper middle‐income country	*Population:* Healthy mothers and infants *Intervention:* Professional BF support interventions and education, including combined professional and peer‐support *Comparator:* Not recorded *Outcomes:* BF duration and exclusivity	Finnish nursing association critique criteria	BFHI is effective so the components should be used in BF interventions Peer support should also be included in BF interventions
Howe‐Heyman and Lutenbacher (2016)	To determine the effectiveness of BFHI to improve BF initiation, duration and exclusivity	4 databases searched English language only Included published and grey literature Searched 1991 to Oct 2014	Studies varied from 113 to 660,355 participants **25 studies in total all BFHI**—1 RCT, remainder observational Range of counties including high, upper middle‐ and lower middle‐income	*Population:* Pregnant or postnatal women delivering in a BFHI hospital, excluding NICU *Intervention:* Hospitals with full BFI accreditation *Comparator:* No control or non‐BFI accredited *Outcomes:* BF initiation, duration and exclusivity	Risk of Bias not explicitly performed States each article was reviewed for BFI adherence, study design, methods, results and limitations	Research with strong methodological design is needed to more conclusively determine the effectiveness of BFI at improving BF rates
Kim et al. (2018)	To review how effectively BF support interventions enable mothers to exclusively BF until 6 months	6 databases, as well as reference lists searched Written in English or Korean Searched Jan 2000 to August 2017	20,825 involved in BFI studies 27 studies in total all RCT, cluster RCT or quasi‐randomised trials (some discrepancies—as also states quasi‐randomised trials were excluded) **4 studies on BFI interventions** BFI studies: Brazil, Belarus, Turkey, Congo	*Population:* Pregnant women and mothers *Intervention:* Strategies or interventions which promote exclusive BF for 6 months, including the BFHI *Comparator:* Standard care/control groups who did not receive interventions to promote exclusive BF for 6 months *Outcomes:* Exclusive BF at 6 months	Cochrane Collaboration's risk of Bias tool	BFI was the most effective subgroup of BF support interventions More rigorous RCT are required, along with economic evaluation to determine the best BF interventions
Munn et al. (2016)	To examine the impact of BFHI in US populations	5 databases searched Searched 2010 to 2015 (plus any articles identified prior to this date through the ancestry method)	Number of participants not documented **16 research articles and 2 policy documents all BFI** US only studies	*Population:* Mothers/infants—included preterm and infants admitted to NICU in the US *Intervention:* BFHI accreditation *Comparator:* Not specified *Outcomes:* Early health outcomes and BF outcomes related to baby‐friendly practices in U.S. settings	Classified using the OXFORD scale for levels of evidence for empirical studies and Melnyk and Fineout‐Overholt ranking scale for qualitative studies	Evidence supports BFI as good practice, however causal mechanisms unclear Further studies of infant outcomes related to BFHI required, especially prospective studies and exploration of maternal experiences
Pérez‐Escamilla et al. (2016)	To examine the impact of BFI on BF and child health outcomes	6 databases, as well as reference lists searched English, Spanish or Portuguese language Searched inception to Dec 2012	Studies varied from 75 to 464,246 women **58 studies in total, all BFI** 2 RCT's, 6 follow up RCTs, the remainder observational 19 countries from South America, North America, Western Europe, Eastern Europe, South Asia, Eurasia and Sub‐Saharan Africa	*Population:* Women with a healthy, full‐term infant in a hospital or birthing centre *Intervention:* BFHI. Women exposed to at least 3 BFI steps, infants born in a BFI designated centre where women exposed to BFHI steps and/or BFCI or other community support *Comparator:* Women not exposed to the BFHI steps *Outcomes:* Exclusive BF at discharge—6 months. Any BF at discharge −12 months. Onset of lactation. Infant health outcomes	A modified grading of recommendations assessment, development and evaluation (GRADE) method	BF outcomes improved by BFI Community support vital for long‐term BF outcomes More high quality RCT are required to better evaluate outcomes
Renfrew et al. (2009, 2010)	To evaluate the effectiveness of clinical, public health and health promotion interventions to promote or inhibit provision of breastmilk to infants in neonatal units	19 databases including trial registers, as well as reference lists searched. 6 additional databases searched for economic evaluation Liaison with advisory group members No language or country of origin limitations Searched inception to Feb 2008	722 for BFI outcomes 48 studies in total: RCT and observational studies **4 studies in BFI subgroup**—all observational 17 countries (11 developing and 6 industrialised)	*Population:* Infants, or mothers of infants, admitted to neonatal units; and those linked to them including fathers/partners, other family members or health professionals *Intervention:* Any intervention addressing BF/feeding with breastmilk in neonatal units or following discharge *Comparator:* Any form of control group *Outcomes:* Measures of BF/breastmilk feeding. Secondary outcomes included clinical/health, process, psychosocial and cost‐effectiveness outcomes	Based on the NICE guidance development methodology and the Cochrane handbook	BF or breastmilk provision is enhanced by skin‐to‐skin contact, effective milk expression, peer support, staff training and BFI accreditation of the maternity hospital
Sinha et al. (2015)	To provide evidence of the effect of interventions on early initiation, exclusive and any BF rates in 5 different settings	3 databases as well as references lists searched. Citation list for included studies searched No language restrictions Searched inception—Oct 2014	Number of participants not recorded 195 studies: RCTs and observational studies **24 were BFI studies**—1 RCT remainder observational studies Countries of origin not recorded	*Population:* Pregnant women and mothers *Intervention:* Interventions to improve BF, delivered to families, community, health staff or other stakeholders. Health systems subgroup included BFHI. Included interventions in preterm infants or babies in NICU *Comparator:* Control groups (not specified) *Outcomes:* Initiation, duration and exclusive BF rates	Cochrane risk of bias assessment tool	Baby friendly hospital support was the most effective intervention to improve rates of any BF
Sinha et al. (2017)	To update evidence on the effect of interventions on early initiation, exclusive and continued BF in LMIC	3 databases as well as references lists searched. Citation list for included studies searched No language restrictions Searched inception—July 2016	Number of participants not recorded 61 studies in total—RCTs and observational studies **10 were BFI studies**—2 RCT, remainder observational studies LMIC but not reported individually which countries	*Population:* Pregnant women and mothers in LMIC countries *Intervention:* Interventions to promote BF conducted in LMIC. 3 major categories according to their setting: Health systems, home and community environments, or a combination of these. Health systems and services included BFHI interventions *Comparator:* Control groups not specified *Outcomes:* Early initiation of BF, exclusive and continued BF	Cochrane risk of bias assessment tool for individual studies and the GRADE approach for pooled estimates from RCTs	BF interventions can improve BF rates in LMIC

Abbreviations: BF, breastfeeding; BFCI, Baby Friendly community Initiative; BFHI, Baby Friendly Hospital Initiative; BFI, Baby Friendly Initiative; LMIC, low‐, middle‐income countries; NICU, neonatal intensive care units; RCT, randomised controlled trial; UK, United Kingdom; US, United States.

### 3.2 Methodological quality of included systematic reviews

Table 3 provides AMSTAR‐2 quality assessment results for each included systematic review.

**TABLE 3:** AMSTAR‐ 2 checklist assessment for each included systematic review

Item Study	1	2^a^	3	4^a^	5	6	7^a^	8	9^a^		10	11^a^		12^a^	13^a^	14^a^	15^a^	16	Confidence in the results of the review
									RCT	NRSI		RCT	NRSI						
Atchan et al. (2013)	X	X	X	/	X	X	X	X	X	X	X	NA	NA	NA	✓	✓	NA	X	Critically low
Beake et al. (2012) Beake et al. (2011)	✓	X	✓	✓	✓	✓	✓	/	✓	✓	X	NA	NA	NA	✓	✓	NA	✓	Low
Chung et al. (2008)	✓	X	X	/	X	✓	/	/	✓	‐	X	✓	‐	✓	✓	✓	X	✓	Critically low
Fairbank et al. (2000)	✓	/	✓	✓	✓	✓	✓	✓	✓	/	X	NA	NA	NA	✓	✓	NA	✓	High
Fallon et al. (2019)	✓	/	✓	/	✓	✓	/	/	‐	✓	X	NA	NA	NA	✓	✓	NA	✓	High
Feltner et al. (2018)	✓	✓	✓	✓	✓	✓	✓	/	✓	✓	X	NA	NA	NA	✓	✓	NA	✓	High
Hannula et al. (2008)	✓	X	X	X	✓	X	X	X	X	X	X	NA	NA	NA	X	✓	NA	X	Critically low
Howe‐Heyman and Lutenbacher (2016)	✓	X	X	/	X	X	X	/	X	X	X	NA	NA	NA	✓	✓	NA	✓	Critically low
Kim et al. (2018)	✓	X	X	X	✓	✓	/	X	✓	‐	X	✓	‐	X	✓	✓	X	✓	Critically low
Munn et al. (2016)	✓	X	✓	X	X	X	X	X	X	X	X	NA	NA	NA	✓	✓	NA	✓	Critically low
Pérez‐Escamilla et al. (2016)	✓	X	✓	/	✓	X	X	✓	✓	✓	X	NA	NA	NA	✓	✓	NA	✓	Critically low
Renfrew et al. (2010) Renfrew et al. (2009)	✓	X	✓	✓	✓	✓	✓	✓	✓	✓	X	NA	NA	NA	✓	✓	NA	✓	Low
Sinha et al. (2017)	✓	/	✓	/	✓	✓	X	X	✓	/	X	✓	✓	✓	✓	✓	X	✓	Critically low
Sinha et al. (2015)	✓	X	✓	/	✓	✓	/	X	✓	/	X	✓	✓	✓	✓	✓	X	✓	Critically low

Item 1: research question; Item 2: protocol development; Item 3: included study design explained; Item 4: comprehensive literature search; Item 5: study selection in duplicate; Item 6: data extraction in duplicate; Item 7: list of excluded studies; Item 8: included study description; Item 9: risk of bias assessment; Item 10: sources of funding of included studies; Item 11: appropriate methods for statistically combining results; Item 12: risk of bias impact on meta‐analysis considered; Item 13: risk of bias considered when interpreting/discussing results; Item 14: heterogeneity of included studies discussed; Item 15: publication bias assessment and Item 16: author conflict of interest.

Abbreviations: NA, no meta‐analysis; NRSI, non‐randomised study of intervention; RCT, randomised controlled trial; X, not met; /, partial yes; ✓, full yes; ‐, this type of study not included.

^a^ Domains considered as critical.

Only one review (Atchan et al., 2013) was judged not to have a clearly focussed research question and inclusion criteria. One review (Feltner et al., 2018) reported a registered review protocol established prior to undertaking the review, and a further three reviews mentioned at least some aspects of a protocol (Fairbank et al., 2000; Fallon et al., 2019; Sinha et al., 2017). The remaining reviews made no explicit reference to a review protocol.

The search strategy was judged to be inadequate in three reviews (Hannula et al., 2008; Kim et al., 2018; Munn et al., 2016), due to no clear justification for the date restrictions applied within their search. A further seven reviews (Atchan et al., 2013; Chung et al., 2008; Fallon et al., 2019; Howe‐Heyman & Lutenbacher, 2016; Pérez‐Escamilla et al., 2016; Sinha et al., 2015, 2017) were judged to only partially meet the comprehensive literature search criteria. The majority of these reviews did not report searching trial/study registers (Atchan et al., 2013; Fallon et al., 2019; Howe‐Heyman & Lutenbacher, 2016; Pérez‐Escamilla et al., 2016; Sinha et al., 2015, 2017); four did not report consulting with experts in the field (Chung et al., 2008; Howe‐Heyman & Lutenbacher, 2016; Sinha et al., 2015, 2017) and three did not explicitly report that attempts were made to search for grey literature (Atchan et al., 2013; Chung et al., 2008; Fallon et al., 2019). It was also unclear in two reviews whether the search was undertaken within 24 months of review completion (Atchan et al., 2013; Pérez‐Escamilla et al., 2016), and one review did not report searching the references list of relevant articles (Howe‐Heyman & Lutenbacher, 2016).

Four reviews (Atchan et al., 2013; Chung et al., 2008; Howe‐Heyman & Lutenbacher, 2016; Munn et al., 2016) did not report authors performing study selection in duplicate and five reviews (Atchan et al., 2013; Hannula et al., 2008; Howe‐Heyman & Lutenbacher, 2016; Munn et al., 2016; Pérez‐Escamilla et al., 2016) did not report undertaking data extraction in duplicate. Only four reviews (Beake et al., 2012; Fairbank et al., 2000; Feltner et al., 2018; Renfrew et al., 2010) provided references and reasons for exclusion of all articles at full text, with a further four reviews (Chung et al., 2008; Fallon et al., 2019; Kim et al., 2018; Sinha et al., 2015) providing the reasons for excluding full text citations without providing individual references.

Only three reviews (Fairbank et al., 2000; Pérez‐Escamilla et al., 2016; Renfrew et al., 2010) were judged to have provided detailed characteristics of the studies included within their review. Four reviews (Atchan et al., 2013; Hannula et al., 2008; Howe‐Heyman & Lutenbacher, 2016; Munn et al., 2016) did not adequately report assessing risk of bias within included studies, and the assessment of risk of bias in non‐randomised studies was deemed to have only partially used a satisfactory technique within three reviews (Fairbank et al., 2000; Sinha et al., 2015, 2017). Of the four reviews that included meta‐analysis (Chung et al., 2008; Kim et al., 2018; Sinha et al., 2015, 2017), all justified the use of meta‐analysis, used appropriate methods and explored the causes of heterogeneity within the results. However, none reported carrying out adequate investigations to determine the impact of potential publication bias on the results and one (Kim et al., 2018) did not report assessing the impact of risk of bias within the included studies on the meta‐analysis results, for example, through sensitivity analysis.

One review (Hannula et al., 2008) did not discuss the likely impact of risk of bias within included studies when interpreting or discussing the review's results. All reviews were judged to have provided at least some exploration, explanation or discussion around heterogeneity of studies included within their review.

All but two reviews (Atchan et al., 2013; Hannula et al., 2008) declared any conflicts of interest for the systematic review; however, none of the studies reported on the funding sources of the included studies within the review.

Overall confidence in the results of the review was rated as high for three reviews (Fairbank et al., 2000; Fallon et al., 2019; Feltner et al., 2018) that only had one or no weaknesses in non‐critical domains, low for two reviews (Beake et al., 2012; Renfrew et al., 2010) that had a weakness within one critical domain and critically low for the remaining nine reviews that had weaknesses within more than one critical domain. No reviews were rated as moderate.

### 3.3 Quality of the studies included within the reviews

**FIGURE 1:**
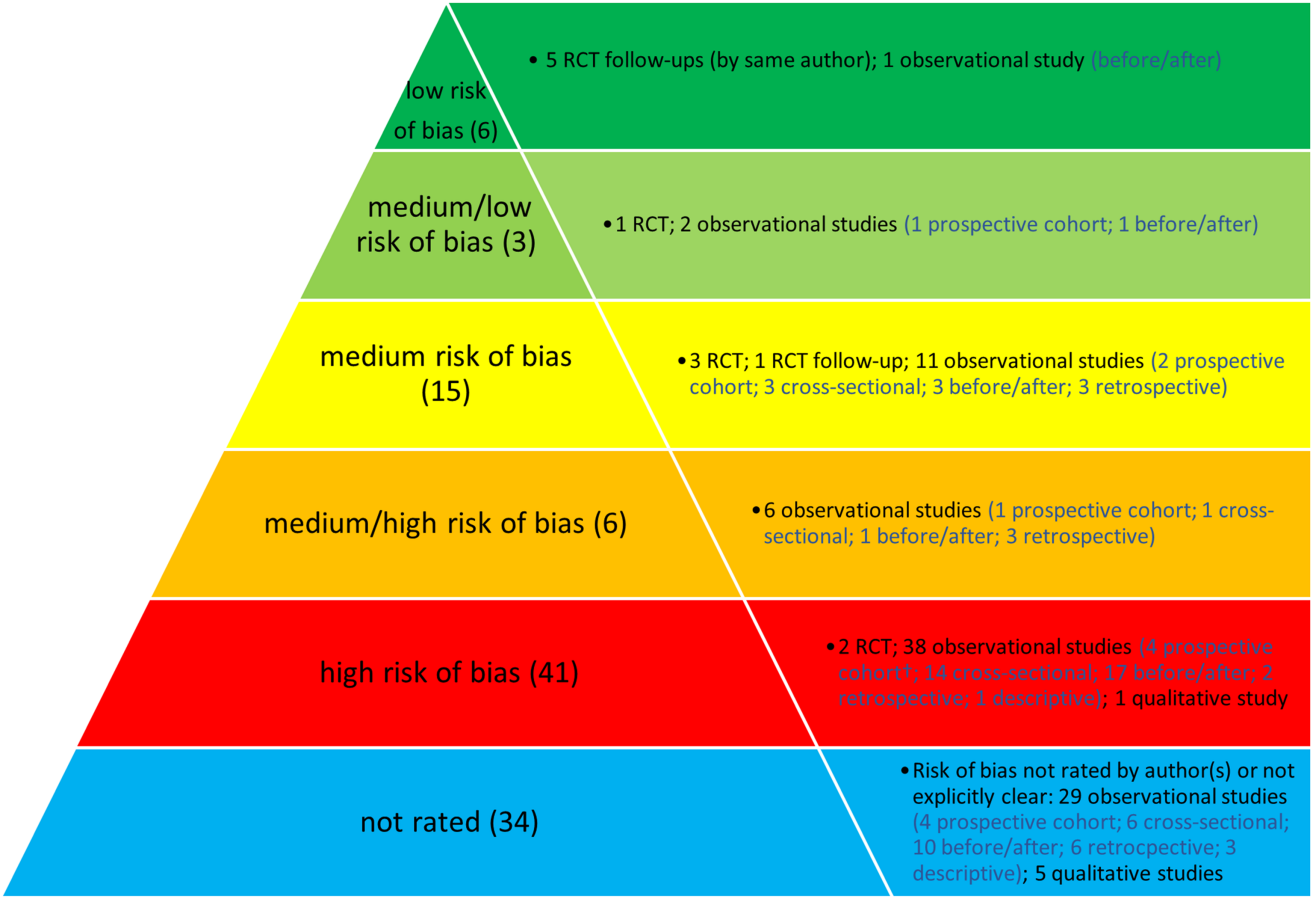
Risk of bias and study design as assessed by review author(s) of the 105 individual studies included within the reviews. ^†^One prospective cohort was non‐comparative

As well as assessing the quality of the included reviews, it was important to consider the quality of the studies included within the reviews as indicated in the Risk of Bias assessments by the review authors (Figure 1). With the exception of two reviews (Atchan et al., 2013; Howe‐Heyman & Lutenbacher, 2016), all reviews reported carrying out a Risk of Bias assessment using various tools, for example, the Cochrane Collaboration Risk of Bias tool or the Joanna Briggs Institute methodological quality checklist. Out of the 105 studies included within the 14 reviews, just over one third were not given an individual rating by the authors. However, where no rating for Risk of Bias was given, an explanation of study limitations was often provided (e.g., no inferential statistics, convenience sampling, selection bias and lack of adjustments for covariates).

Figure 1 also provides the study designs included within each risk of bias category. Where there was discrepancy about the design of studies included in multiple reviews, the original study was obtained for clarification. The methodology of some studies however remained ambiguous.

The most cited study was a randomised controlled trial (RCT) conducted in Belarus, appearing in 11 of the 14 reviews (Kramer et al., 2001). This was rated to have medium/low risk of bias.

### 3.4 Outcomes

There was considerable overlap of the studies within the systematic reviews; therefore, where a study was included within multiple reviews, the results were only reported once in order to avoid duplication. Appendix G provides full results from each review.

#### 3.4.1 Breastfeeding intention

No review reported breastfeeding intention as an outcome.

#### 3.4.2 Breastfeeding initiation

Breastfeeding initiation was reported in 11 systematic reviews. The results of four reviews (Chung et al., 2008; Fairbank et al., 2000; Fallon et al., 2019; Hannula et al., 2008) have not been reported narratively to avoid duplication as all of the study(ies) contributing to this outcome within those reviews were already reported within another included review (Beake et al., 2012, Pérez‐Escamilla et al., 2016 or Sinha et al., 2015). The other seven reviews reported outcomes from 32 studies; 17 of which were only reported within one review. No review reported this outcome from any RCT evidence. The majority of the included observational study evidence suggested breastfeeding initiation increased with BFI accreditation; however, this was not consistent across the included studies within all reviews, with some studies showing no difference in breastfeeding initiation with BFI accreditation (Beake et al., 2012; Feltner et al., 2018; Howe‐Heyman & Lutenbacher, 2016).

#### 3.4.3 Exclusive breastfeeding

Exclusive breastfeeding outcomes were reported in 13 systematic reviews. All of the studies within seven reviews (Beake et al., 2012; Chung et al., 2008; Fallon et al., 2019; Feltner et al., 2018; Hannula et al., 2008; Kim et al., 2018; Sinha et al., 2015) were either reported in another review (Atchan et al., 2013; Munn et al., 2016; Pérez‐Escamilla et al., 2016 or Sinha et al., 2017) or their unique studies were not evaluating full BFI accreditation. The other reviews reported a total of 56 studies, of which 37 were only reported within one included review. Most, but not all, RCT and observational study evidence showed improved exclusive breastfeeding with BFI intervention when evaluated at different time points including at 2 weeks (Renfrew et al., 2010), hospital discharge for neonates admitted to NICU (Renfrew et al., 2010), up to 1 month (Howe‐Heyman & Lutenbacher, 2016; Sinha et al., 2017), 1 to 5 months (Sinha et al., 2017) and any time points up to 6 months (Pérez‐Escamilla et al., 2016) or over 1 month (Howe‐Heyman & Lutenbacher, 2016) and at unclear time points (Atchan et al., 2013; Munn et al., 2016). This outcome was difficult to interpret however due to different definitions of exclusive breastfeeding within each study and review.

Evidence from three RCTs and some low‐quality observational studies suggested good implementation of Step 10 (continued support after discharge, e.g., home peer support) was necessary for improvements in both exclusive or any breastfeeding (Pérez‐Escamilla et al., 2016).

#### 3.4.4 Any breastfeeding

Thirteen reviews reported duration or rates of any breastfeeding. All of the included studies within five reviews (Chung et al., 2008; Fairbank et al., 2000; Hannula et al., 2008; Renfrew et al., 2010; Sinha et al., 2017) were either reported in another review (Pérez‐Escamilla et al., 2016, or Sinha et al., 2015) or were not studies evaluating full BFI accreditation. Reviews included a total of 60 studies, of which 37 were only incorporated into one review. Most RCT and observational studies showed improved rates of any breastfeeding in groups receiving BFI interventions when evaluated at different time points including 1 week (Beake et al., 2012), 2 weeks (Beake et al., 2012; Fallon et al., 2019), 1 month (Beake et al., 2012; Howe‐Heyman & Lutenbacher, 2016), over 1 month (Howe‐Heyman & Lutenbacher, 2016), 6 weeks (Atchan et al., 2013), 2 months, 3 to 4 months and 6 months (Beake et al., 2012), up to 6 months (Sinha et al., 2015) at any point up to 12 months (Feltner et al., 2018; Pérez‐Escamilla et al., 2016) and at an unclear time point (Munn et al., 2016). The rate of continued breastfeeding from 6 to 23 months was no different between intervention and control groups (Sinha et al., 2015).

There was some evidence that the more BFHI steps that were implemented, the longer the duration of any breastfeeding (Atchan et al., 2013; Feltner et al., 2018; Pérez‐Escamilla et al., 2016), with women who receive no BFHI practices 13 times more likely to discontinue breastfeeding prior to 6 months (Atchan et al., 2013). There was some evidence that implementation of Steps 2 (staff training) and 4 (supporting mothers to initiate and maintain breastfeeding) may be particularly important (Fallon et al., 2019).

#### 3.4.5 Health outcomes

Only two observational studies reported maternal health outcomes. One study found women delivering in a BFI hospital were less likely to experience mastitis, and the other study showed that women were more likely to remain amenorrhoeic at 6 months when giving birth in a unit with BFI accreditation. Five systematic reviews reported infant outcomes. Four of these (Atchan et al., 2013; Beake et al., 2012; Chung et al., 2008; Pérez‐Escamilla et al., 2016) all reported the results from the same medium‐low risk of bias RCT (Kramer et al., 2001) which showed decreased gastrointestinal infection and allergic dermatitis in infants, but no differences in respiratory tract infections, infant weight or head circumference measurements at 1 year of age. Long‐term outcomes up to 6.5 years of age showed increases in neurodevelopment, but no differences in childhood obesity, blood pressure measurements, incidence of allergies or asthma, dental health or child behaviour. The final systematic review (Munn et al., 2016) stated that there was limited evidence from three studies to determine the impact of BFHI on infant health outcomes and that the health outcomes for late preterm infants was not clear.

#### 3.4.6 Other outcomes

Four reviews reported other outcomes. Maternal knowledge about breastfeeding was higher in women receiving a BFI intervention than a control group and women in BFI intervention groups reported increased breastfeeding support in hospital (Beake et al., 2012). Thematic synthesis of women's experiences across five qualitative studies within one review (Fallon et al., 2019) showed that professional support was highly influential in women's experiences of BFI care; BFI may promote unrealistic expectations and not meet women's individual needs and can have an emotional impact on women especially guilt and feeling pressurised to breastfeed.

Compared to those in control groups, healthcare professionals receiving BFHI training have increased knowledge of the BFI (Beake et al., 2012; Fairbank et al., 2000), were more likely to intend to change their practice (Fairbank et al., 2000) and were more likely to comply with BFHI practices and philosophy within both qualitative and quantitative studies (Munn et al., 2016).

### 3.5 Subgroup analysis

A subgroup analysis was planned according to income level of the country of the original studies. One review (Sinha et al., 2015) presented results according to country income level across all included interventions, but not for BFI accreditation specific interventions. Four reviews with critically low confidence in the findings (Atchan et al., 2013; Chung et al., 2008;Kim et al., 2018 ; Sinha et al., 2017) and one review with high confidence in the findings (Fairbank et al., 2000) either specifically reported the impact of BFI accreditation in low‐ and middle‐income countries (LMIC) or only included BFHI studies from these countries. The reviews, including a total of 14 studies, found BFHI accreditation increased breastfeeding initiation, exclusive or predominant breastfeeding at time points up to 6 months (Atchan et al., 2013; Chung et al., 2008; Fairbank et al., 2000; Kim et al., 2018; Sinha et al., 2017), continued breastfeeding up to 23 months (Sinha et al., 2017) and reduced gastrointestinal infection and atopic dermatitis in infants (Chung et al., 2008). BFHI was viewed to have ‘immense potential’ to support breastfeeding in LMIC, as could education and counselling interventions (Sinha et al., 2017). However, more research is needed due to limited current evidence (Kim et al., 2018; Sinha et al., 2017), and the currently available data had frequently not been collected for research purposes (Atchan et al., 2013).

Three reviews were either solely focussed on high‐income countries or looked at these as a subgroup. Two reviews with critically low confidence in the findings looked at a total of 23 studies from the United States (US; Munn et al., 2016; Pérez‐Escamilla et al., 2016). US studies were believed to support the effectiveness of BFI implementation on breastfeeding initiation and exclusivity; however, none were high quality studies, none examined child health outcomes (Munn et al., 2016; Pérez‐Escamilla et al., 2016), there were concerns over inconsistencies in reporting breastfeeding duration rates and no studies showed an impact in rural areas within the US (Munn et al., 2016). The final review (Fallon et al., 2019) with high confidence in the findings included six quantitative studies undertaken in the United Kingdom (UK). Studies suggested an increase in initiation in hospitals with BFHI accreditation. However, there were no differences in initiation between services with BFHI accreditation and those with a certificate of commitment (Fallon et al., 2019). Increased exclusive breastfeeding rates up to 6 weeks were seen across the different studies in units with BFHI accreditation or a certificate of commitment, but no differences in any breastfeeding by 4 weeks were noted with either BFHI accreditation of a certificate of commitment. Three studies of BFCI accreditation found positive effect on any breastfeeding at 6–8 weeks.

No reviews reported stage of BFI accreditation, so a subgroup analysis on this aspect could not be performed.

### 3.6 GRADE summary of findings

Table 4 presents a summary of findings. For all four outcomes breastfeeding initiation, exclusive breastfeeding, any breastfeeding and health outcomes, confidence in the current evidence was judged to be very low.

**TABLE 4:** Summary of findings and overall certainty in the evidence regarding the impact of baby friendly initiative accreditation on breastfeeding and infant health outcomes

Certainty assessment							Summary of findings
Systematic reviews	Risk of bias	Inconsistency	Indirectness	Imprecision	Publication bias	Overall certainty of evidence	
Breastfeeding initiation							
7 systematic reviews	Very serious^a^ ^,^ ^b^	Serious^c^	Serious^d^	Serious^e^	None	⊕◯◯◯VERY LOW	Most evidence (randomised trails, quasi experimental and observational) suggests an increase in breastfeeding initiation with BFI accreditation
Exclusive breastfeeding							
9 systematic reviews	Very serious^a^ ^,^ ^b^	Serious^c^	Serious^f^	Serious^e^	None	⊕◯◯◯VERY LOW	Most but not all evidence (randomised trails, quasi experimental and observational) suggests an improved rates of exclusive breastfeeding with BFI accreditation when evaluated at different timepoints up to 6 months
Any breastfeeding							
8 systematic reviews	Very serious^a^ ^,^ ^b^	Serious^c^	Serious^g^	Serious^e^	None	⊕◯◯◯VERY LOW	Most studies (randomised trails, quasi experimental and observational) suggest an increase in any breastfeeding with BFI accreditation when evaluated at different time points up to 23 months.
Infant health outcomes							
2 systematic reviews	Serious^h^	Not applicable	Serious^i^	Serious^j^	None	⊕◯◯◯VERY LOW	One RCT and its associated follow‐up studies showed decreased gastrointestinal infection and allergic dermatitis in infants and improved neuro development at 6.5 years, but no difference in other infant or child health outcomes Three other observational studies provided limited evidence regarding the impact of BFHI on early infant outcomes including infants in NICU or preterm

*Note*: ‘Summary of Findings’ table was produced using the GRADE approach (Schünemann et al., 2013).

^a^ Downgraded (−1) for risk of bias as most included studies for this outcome were medium or high risk of bias and overall confidence in the results of most of the reviews contributing to this outcome was judged to be either low or critically low.

^b^ Downgraded (−1) for risk of bias as most included studies for this outcome were of poor methodological design.

^c^ Downgraded (−1) for inconsistency because when considered, heterogeneity was high between studies.

^d^ Downgraded (−1) for indirectness because not all included interventions within systematic reviews measured the impact of full BFI accreditation and due to lack of a consistent definition of breastfeeding initiation between included studies.

^e^ Downgraded (−1) for imprecision due to lack of clarity over the size of the effect.

^f^ Downgraded (−1) for indirectness because not all included interventions within systematic reviews measured the impact of full BFI accreditation and due to lack of a consistent definition of exclusive breastfeeding between included studies.

^g^ Downgraded (−1) for indirectness because not all included interventions within systematic reviews measured the impact of full BFI accreditation and due to lack of a consistent definition of any breastfeeding between included studies.

^h^ Downgraded (−1) for risk of bias as confidence in the findings of the reviews which contributed to this outcome was judged to be crucially low.

^i^ Downgraded (−1) for indirectness as most studies were regarding infants who required neonatal admission or were preterm, few studies looked at health outcomes in all infants.

^j^ Downgraded (−1) for imprecision due to few studies, providing limited evidence regarding the long‐term impact of BFI accreditation on infant health.

All outcomes were predominantly downgraded for risk of bias, indirectness and imprecision. Within this overview, outcomes were downgraded either −1 or −2 for risk of bias due to the overall confidence in the results of the majority of the included systematic reviews from the AMSTAR‐2 checklist was either low or critically low and due to the poor methodological design of many studies, as most of the included studies had been rated as medium or high risk of bias by review authors. The evidence was downgraded for indirectness as not all included studies within the reviews evaluated the impact of full BFI accreditation, and there was lack of consistency in definitions of initiation, exclusivity and any breastfeeding. Infant health outcomes were downgraded for indirectness as several studies focussed exclusively on infants who were preterm or required neonatal admission. The evidence was downgraded for imprecision as there is currently a lack of certainty over the size of the effect and for infant health outcomes due to very few studies providing evidence regarding the long‐term impact of BFI accreditation on infant health. The outcomes of initiation, exclusivity and any breastfeeding were downgraded for inconsistency due to heterogeneity being high between studies within the included reviews.

## 4 DISCUSSION

The results of this overview suggest that BFI accreditation may improve breastfeeding initiation and duration of exclusive and any breastfeeding, although no differences were seen within one review investigating longer term breastfeeding between 6 and 23 months. There is limited current evidence around the impact of BFI on maternal and infant health outcomes. Confidence in this evidence was however judged to be very low for all outcomes.

### 4.1 Impact of BFI

Breastfeeding intention was not reported within any of the reviews, despite this being a crucial factor in breastfeeding initiation, so the impact of BFI accreditation could not be assessed on this outcome. To achieve BFI accreditation, improved initiation rates have to be evidenced; it is therefore unsurprising that studies showed improved initiation rates, as without this accreditation would not have been achieved (Fallon et al., 2019).

Increasing exclusive breastfeeding is viewed as one of the top interventions to assist in achieving many of the Sustainable Development Goals and to reduce under‐5 mortality (Victora et al., 2016). Most reviews found some improvements in exclusivity of breastfeeding; however, the duration of the improvements was much less clear. Similarly, there was lack of clarity over the duration of improvements to the rate of any breastfeeding. Reviews looking at the evidence within high‐income countries such as the US and UK questioned the impact of BFI on long‐term outcomes (Fallon et al., 2019; Pérez‐Escamilla et al., 2016), with community BFI accreditation suggested to lead to changes in the rate of any breastfeeding at 6 to 8 weeks. However, within low‐ and middle‐income settings, improved rates of any breastfeeding with BFI accreditation were seen up to 23 months (Sinha et al., 2017), although the confidence in the findings of this review is critically low.

There was not sufficient evidence reported around long‐term maternal health outcomes, and current evidence of the impact of full BFI accreditation on infant health outcomes was based on just one RCT and a few observational studies which showed some impact on reducing gastrointestinal infection and allergic dermatitis, but not other health outcomes. The RCT (Kramer et al., 2001) was undertaken 20 years ago, and the authors themselves urged caution about generalisation of their results given the extent of changes undertaken within hospitals in Belarus as a result of BFI accreditation and due to the long length of hospital stays in Belarus (6–7 days postpartum) compared to stays of less than 48 h common in many other countries. The authors felt both of these factors could have increased the impact of a hospital‐based intervention within the Belarus context. More comprehensive and robust data on breastfeeding outcomes and their impact on long‐term health outcomes are essential to allow measurement of any sustained effectiveness of BFI accreditation (Eidelman, 2018).

Studies undertaken in countries with different healthcare systems (e.g., length of hospital stay), economic backgrounds, cultures, rates of breastfeeding initiation or rates of exclusive or any breastfeeding at various time points postpartum may respond differently to BFI accreditation (Fallon et al., 2005, 2019). There is therefore a need to consider the cultural, socioeconomic and practice context of the country where each study occurs in comparison to the country where the impact of BFI is to be assessed.

### 4.2 Evidence for the individual BFI components

While evaluation of individual BFI steps was not the focus of this overview, it is important to discuss the impact of individual steps, as the causal mechanism for how BFI practices may improve breastfeeding rates have not been fully identified (Munn et al., 2016). The benefit of some of the individual steps has previously been shown. Step 4, early skin‐to‐skin contact, has been shown to increase breastfeeding rates at 1 to 4 months postpartum (Moore et al., 2016). Community support (part of Step 10) is believed to be essential for long‐term breastfeeding duration improvements (Fallon et al., 2019; Pérez‐Escamilla et al., 2016) as even in countries with high breastfeeding initiation rates, the drop off of exclusive breastfeeding means countries fall far short of the WHO guidance of 6 months (Victora et al., 2016). The impact of Step 3, prenatal breastfeeding education, is unclear with one review stating that prenatal interventions were effective at increasing initiation, exclusivity and duration of breastfeeding although there was lack of clarity over the most effective method of delivery (Wouk et al., 2017) and another review stating there was no conclusive evidence to support antenatal education to improve initiation, exclusivity or duration of breastfeeding (Lumbiganon et al., 2016). Evidence around the impact on breastfeeding outcomes of Step 2—training of health professionals (Gomez‐Pomar & Blubaugh, 2018), Step 6—provision of additional foods or fluids (Smith & Becker, 2016); Step 7—rooming in (Jaafar, Ho, & Lee, 2016) and Step 9—pacifier use (Jaafar, Ho, Jahanfar, & Angolkar, 2016) is currently limited. Furthermore, some have raised concerns around the enhanced risk of sudden unexpected collapse in newborn infants in skin‐to‐skin contact, especially when mothers are tired or sedated immediately after delivery and also over the protective effect against Sudden Infant Death Syndrome (SIDS) of pacifier use among breastfed infants (Gomez‐Pomar & Blubaugh, 2018). Further research is therefore required to identify the effectiveness of individual components as well as full BFI accreditation as a whole.

### 4.3 Cost effectiveness of BFI

The cost of delivering BFI was not an outcome assessed within any of the included systematic reviews. A systematic review that did not meet the criteria for inclusion within this overview (Carroll et al., 2020) however identified three studies that had assessed the cost of implementing BFI at hospital level. All three studies were undertaken in the US. Two studies found that the cost per delivery was increased but that this was not significant, being $35 higher within BFI accredited units than in non‐BFI accredited units (DelliFraine et al., 2011) and $19 more in a hospital implementing 6–8 BFI steps than in a hospital implementing 3–5 BFI steps (Allen et al., 2013). However, the first of these studies although matching BFI units to a non‐BFI unit within the same city did not match for deprivation, education level or ethnicity, all of which may impact upon costs. It also did not calculate upfront costs such as training and the cost of accreditation (DelliFraine et al., 2011). The final study assessed costs within one maternity unit, alongside interviews and an online survey with other baby friendly hospitals. They found each birth increased in cost by $148 with BFI accreditation (DelliFraine et al., 2013). The majority of this increase was due to the costs of having to pay for infant formula due to the prevention of free or heavily discounted infant formula milk being provided to BFI accredited units; therefore, as exclusive breastfeeding increases, these costs would be expected to decrease over time. In countries where hospitals already pay market value for infant formula, these costs are therefore likely to be less.

BFI has been important in raising the profile of breastfeeding globally. However, the evidence currently is not sufficient to proclaim BFI as a superior intervention to all other breastfeeding interventions. The effectiveness of BFI is especially questioned in areas where evidence‐based practices to support breastfeeding already exist (Brodribb et al., 2013). Although it is likely that benefits are incurred as a result of BFI accreditation, these are currently poorly quantified, and superiority comparisons with other breastfeeding interventions need further investigation. Rigorous evaluation is particularly required to compare the cost‐effectiveness of BFI compared to other structured interventions.

### 4.4 Limitations

The main limitation of this overview is the limited quality of the included reviews and studies. The majority of current evidence is based on before‐after studies or cohort studies which did not control for other confounding factors.

It is recognised that not all of the studies incorporated into the included systematic reviews were full BFI accreditation studies with some including studies of individual steps or various combinations of BFI steps. One review also incorporated other ‘structured support’ interventions alongside BFI interventions.

When judging the quality of included reviews, the authors were only able to assess the information contained within the published articles, alongside some basic searches for review protocols. However, due to word limitations, it is recognised that the published information may not fully reflect all of the processes undertaken within a review.

### 4.5 Implications

Currently, there is a lack of clear evidence around long‐term improved duration of breastfeeding and health benefits of BFI, particularly within high‐income countries. Caution is required when determining the potential impact of BFI accreditation in different global contexts using currently available evidence.

Qualitative evidence around BFI has found women to describe that their reality of breastfeeding differed from their expectations and that they can feel pressurised to breastfeed or guilt when unable to succeed in hospitals with BFI accreditation (Fallon et al., 2019). Woman‐centred approaches are increasingly recognised as important, with a need for research to explore the acceptability of the revised 10 steps to parents across a range of international contexts (Aryeetey & Dykes, 2018) and the need to address the growth of cultural acceptance of formula feeding within many countries.

## 5 CONCLUSIONS

This overview suggests that there may be some improvement in initiation and breastfeeding duration from Baby Friendly Initiative accreditation especially in low‐income countries, although the duration of any improvements in breastfeeding is uncertain and confidence in these findings was very low due to the poor methodological quality of existing evidence. Evidence around the impact of BFI accreditations on long‐term health of mothers and babies is currently minimal. Well‐designed controlled trials are required to better evaluate the short‐ and long‐term impact of BFI accreditation.

## CONFLICTS OF INTEREST

The authors have no competing interests to declare.

## CONTRIBUTIONS

FF, AM and HS developed the protocol; FF and AM screened titles, abstracts and full texts against inclusion criteria; FF and AM assessed risk of bias; HS resolved any disagreement over inclusion and risk of bias assessments; FF and AM extracted data; FF, AM and HS performed the analysis; FF drafted the manuscript; AM and HS revised the manuscript and FF, AM and HS agreed the final manuscript.

## Data Availability

Data sharing is not applicable to this article as no new data were created or analysed in this study.

## APPENDIX A TEN STEPS TO SUCCESSFUL BREASTFEEDING (UNITED NATIONS CHILDREN'S FUND & WORLD HEALTH ORGANIZATION, 2018)

1a. Comply fully with the *International Code of Marketing of Breast‐milk Substitutes* and relevant World Health Assembly resolutions.

1b. Have a written infant feeding policy that is routinely communicated to staff and parents.

1c. Establish ongoing monitoring and data‐management systems.

2. Ensure that staff have sufficient knowledge, competence and skills to support breastfeeding.

3. Discuss the importance and management of breastfeeding with pregnant women and their families.

4. Facilitate immediate and uninterrupted skin‐to‐skin contact and support mothers to initiate breastfeeding as soon as possible after birth.

5. Support mothers to initiate and maintain breastfeeding and manage common difficulties.

6. Do not provide breastfed newborns any food or fluids other than breast milk, unless medically indicated.

7. Enable mothers and their infants to remain together and to practise rooming‐in 24 hours a day.

8. Support mothers to recognise and respond to their infants' cues for feeding.

9. Counsel mothers on the use and risks of feeding bottles, teats and pacifiers.

10. Coordinate discharge so that parents and their infants have timely access to ongoing support and care.

United Nations Children's Fund & World Health Organization (2018). Protecting, promoting and supporting breastfeeding in facilities providing maternity and newborn services: The revised Baby‐Friendly hospital initiative. Geneva: UNICEF and WHO.

## APPENDIX B SEVEN‐POINT BABY FRIENDLY INITIATIVE FOR SUSTAINED BREASTFEEDING IN THE COMMUNITY (UNICEF, 2014)

1. Have a written breastfeeding policy that is routinely communicated to all healthcare staff.
2. Train all staff involved in the care of mothers and babies in the skills necessary to implement the policy.
3. Inform all pregnant women about the benefits and management of breastfeeding.
4. Support mothers to initiate and maintain breastfeeding.
5. Encourage exclusive and continued breastfeeding, with appropriately‐timed introduction of complementary foods.
6. Provide a welcoming atmosphere for breastfeeding families.
7. Promote co‐operation between healthcare staff, breastfeeding support groups and the local community.

UNICEF (2014). Baby Friendly—7 point plan for sustaining breastfeeding in the community. Retrieved from https://www.unicef.org.uk/babyfriendly/wp-content/uploads/sites/2/2014/02/7_point_plan_community.pdf.

## APPENDIX C EXAMPLE OF THE SEARCH STRATEGY IN CINAHL (VIA EBSCO HOST)

1. baby friendly hospital initiative
2. baby friendly initiative
3. UNICEF breastfeeding initiative
4. baby friendly accredit*
5. baby friendly
6. 1 OR 2 OR 3 OR 4 OR 5
7. systematic review
8. meta‐analysis
9. review of the literature
10. literature review
11. meta‐analysis
12. review
13. (MH “Systematic review”)
14. 7 OR 8 OR 9 OR 10 OR 11 OR 12 OR 13
15. 6 AND 14

## APPENDIX D AMSTAR‐2 QUALITY APPRAISAL TOOL (Shea et al., 2017)

**Table:**

**1. Did the research questions and inclusion criteria for the review include the components of PICO?** □ Population □ Intervention □ Comparator group □ Outcome GWG **□ Yes □ No**
**2. Did the report of the review contain an explicit statement that the review methods were established prior to the conduct of the review and did the report justify any significant deviations from the protocol?** For Partial Yes: The authors state that they had a written protocol or guide that included ALL the following: □ review question(s) □ a search strategy □ inclusion/exclusion criteria □ a risk of bias assessment For Yes: As for partial yes, plus the protocol should be registered and should also have specified: □ a meta‐analysis/synthesis plan, if appropriate, □ a plan for investigating causes of heterogeneity □ justification for any deviations from the protocol **□ Yes □ Partial Yes □ No**
**3. Did the review authors explain their selection of the study designs for inclusion in the review?** For Yes, the review should provide: □ Explanation for including only RCTs □ OR Explanation for including only NRSI (Non‐Randomised Study of Intervention) □ Explanation for including both RCT and NRSI **□ Yes □ No**
**4. Did the review authors use a comprehensive literature search strategy?** For Partial Yes (all the following): □ searched at least 2 databases (relevant to research question) □ provided key word and/or search strategy □ justified publication restrictions (e.g. language) For Yes, should also have (all the following): □ searched the reference lists/bibliographies of included studies ( □ searched trial/study registries □ included/consulted content experts in the field □ where relevant, searched for grey literature □ conducted search within 24 months of completion of the review **□ Yes □ Partial Yes □ No**
**5. Did the review authors perform study selection in duplicate?** For Yes, either ONE of the following: □ at least two reviewers independently agreed on selection of eligible studies and achieved consensus on which studies to include □ OR two reviewers selected a sample of eligible studies and achieved good agreement (at least 80%), with the remainder selected by one reviewer. **□ Yes □ No**
**6. Did the review authors perform data extraction in duplicate?** For Yes, either ONE of the following: □ at least two reviewers achieved consensus on which data to extract from included studies □ OR two reviewers extracted data from a sample of eligible studies and achieved good agreement (at least 80%), with the remainder extracted by one reviewer. **□ Yes □ No**
**7. Did the review authors provide a list of excluded studies and justify the exclusions?** For Partial Yes: □ provided a list of all potentially relevant studies that were read in full‐text form but excluded from the review For Yes, must also have: □ Justified the exclusion from the review of each potentially relevant study **□ Yes □ Partial Yes □ No**
**8. Did the review authors describe the included studies in adequate detail?** For Partial Yes (ALL the following): **□** described populations □ described interventions **□** described comparators □ described outcomes □ described research designs For Yes, should also have ALL the following: □ described population in detail □ described intervention in detail (including doses where relevant) □ described comparator in detail (including doses where relevant) □ described study's setting □ timeframe for follow‐up **□ yes □ partial yes □ no**
**9. Did the review authors use a satisfactory technique for assessing the risk of bias (RoB) in individual studies that were included in the review?** **RCTs** For Partial Yes, must have assessed RoB from □ unconcealed allocation □ lack of blinding of patients and assessors when assessing outcomes (unnecessary for objective outcomes such as all cause mortality) For Yes, must also have assessed RoB from: □ allocation sequence that was not truly random, □ selection bias **□ Yes □ Partial Yes □ No □ Includes only NRSI** **NRSI** For Partial Yes, must have assessed RoB from □from confounding *and* □from selection bias For yes, must also have assessed RoB from: □methods used to ascertain exposures and outcomes *and* □selection of the reported result from among multiple measurements or analyses of the specified outcome **□yes □partial yes □no □includes only RCT**
**10. Did the review authors report on the sources of funding for the studies included in the review?** For Yes □ Must have reported on the sources of funding for individual studies included in the review. Note: Reporting that the reviewers looked for this information but it was not reported by study authors also qualifies **□ Yes □ No**
**11. If meta‐analysis was performed did the review authors use appropriate methods for statistical combination of results?** **RCT** For Yes: □ The authors justified combining the data in a meta‐analysis □ AND they used an appropriate weighted technique to combine study results and adjusted for heterogeneity if present. □ AND investigated the causes of any heterogeneity **□ Yes □ No □ No meta‐analysis conducted** **NRSI** For Yes: □ The authors justified combining the data in a meta‐analysis □ AND they used an appropriate weighted technique to combine study results and adjusted for heterogeneity if present. □ AND they statistically combined effect estimates from NRSI that were adjusted for confounding, rather than combining raw data, or justified raw data when adjusted effect estimates were not available □AND they reported the summary effect estimates for RCTs and NRSI separately when both included in the review **□yes □no □no meta‐analysis conducted**
**12. If meta‐analysis was performed, did the review authors assess the potential impact of RoB in individual studies on the results of the meta‐analysis or other evidence synthesis?** For Yes: □ included only low risk of bias RCTs □ OR, if the pooled estimate was based on RCTs and/or NRSI at variable RoB, the authors performed analyses to investigate possible impact of RoB on summary estimates of effect. **□ Yes □ No □ No meta‐analysis conducted**
**13. Did the review authors account for RoB in individual studies when interpreting/discussing the results of the review?** For Yes: □ included only low risk of bias RCTs □ OR, if RCTs with moderate or high RoB or NRSI were included the review provided a discussion of the likely impact of RoB on the results **□ Yes □ No**
**14. Did the review authors provide a satisfactory explanation for, and discussion of, any heterogeneity observed in the results of the review?** For Yes: □ There was no significant heterogeneity in the results □ OR if heterogeneity was present the authors performed an investigation of sources of any heterogeneity in the results and discussed the impact of this on the results of the review **□ Yes □ No**
**15. If they performed quantitative synthesis did the review authors carry out an adequate investigation of publication bias (small study bias) and discuss its likely impact on the results of the review?** For Yes: □ performed graphical or statistical tests for publication bias and discussed the likelihood and magnitude of impact of publication bias **□ Yes □ No □ No meta‐analysis conducted**
**16. Did the review authors report any potential sources of conflict of interest, including any funding they received for conducting the review?** For Yes: □ The authors reported no competing interests OR □ The authors described their funding sources and how they managed potential conflicts of interest **□ Yes □ No**

*Note*: Shea, B. J., Reeves, B. C., Wells, G., Thuku, M., Hamel, C., Moran, J. … Henry D. A. (2017). AMSTAR 2: A critical appraisal tool for systematic reviews that include randomised or non‐randomised studies of healthcare interventions, or both. *BMJ*, 358, j4008.

## APPENDIX E PRISMA 2009 FLOW DIAGRAM OF STUDY SELECTION

From Moher D, Liberati A, Tetzlaff J, Altman DG, The PRISMA Group (2009). Preferred Reporting Items for Systematic Reviews and Meta‐Analyses: The PRISMA Statement. *PLoS Med* 6(7): e1000097. doi:10.1371/journal.pmed1000097

## APPENDIX F REASONS FOR EXCLUDING STUDIES AT FULL TEXT

**Table:**

Study	Reason
Baby friendly update, 2010	Not BFI accreditation evaluation
Balogun et al., 2016	BFI accreditation outcomes not evaluated as a specific subgroup
Beake, Bick, Narracott, & Chang, 2017	BFI not an intervention evaluated
Britton, McCormick, Renfrew, Wade, & King, 2007	BFI accreditation outcomes not evaluated as a specific subgroup. Review now updated by Renfrew, McCormick, Wade, Quinn, & Dowswell, 2012 and McFadden et al., 2017.
Buccini, Pérez‐Escamilla, Compte, & Flores, 2018	Not full text—protocol only
Carroll et al., 2020	Looks at costs of implementing BFI, not an economic evaluation of BFI accreditation
Cleminson, Oddie, Renfrew, & McGuire, 2015	Not a systematic review
Cramton, Zain‐Ul‐Abideen, & Whalen, 2009	Aims to examine state of breastfeeding in USA, not evaluating BFI accreditation outcomes. Not a systematic review
de Oliveira, Camacho, & Tedstone, 2001	Not an evaluation of BFI accreditation effectiveness
DeMott et al., 2006	Not a systematic review of BFI accreditation outcomes
Dyson, Renfrew, McFadden, McCormick, Herbert, & Thomas, 2010	Policy and public health recommendations—combined previous systematic reviews with consultation to develop these policy recommendations
Dyson, McCormick, & Renfrew, 2005	BFI accreditation outcomes not evaluated as a specific subgroup
Fallon et al., 2005	Not a systematic review
Figueredo, Mattar & De Vilhena Abrão, 2012	Evaluates individual steps, not BFI accreditation overall
Forster & McLachlan, 2007	Does not evaluate overall BFI accreditation as an intervention. Not a systematic review
Gli, Spence, Lynn, Tubman, & Sadeq, 2019	Does not evaluate breastfeeding or other clinical outcomes
Gomez‐Pomar & Blubaugh, 2018	Not a systematic review
Guise et al., 2003	Not an evaluation of BFI accreditation effectiveness
Haroon, Das, Salam, Imdad, & Bhutta, 2013	BFHI include as part of ‘facility based interventions’ not investigated as its own specific subgroup
Health Canada, the Canadian Paediatric Society, Dietitians of Canada, & Breastfeeding Committee for Canada, 2012	Guideline/policy recommendations. Not a systematic review
Lubold, 2017	Not looking at the impact of BFI implementation—looking at BFI as one among other variables that could predict high/low breastfeeding initiation rates. Not a systematic review
Martens, 2012	Not a systematic review—reviews all publications linked to the Kramer et al., 2001 study
McFadden et al., 2017	BFI accreditation outcomes not evaluated as a specific subgroup
Naylor, 2001	Not an evaluation of BFI outcomes
Patnode, Henninger, Senger, Perdue, & Whitlock, 2016	BFI accreditation outcomes not evaluated as a specific subgroup—evaluated alongside other system level support
Philipp & Merewood, 2004	Overview review of BFI development, individual components as well as current evidence
Pramono, Desborough, & Smith, 2019	Looking at policy—not outcomes related to BFI accreditation
Protheroe, Dyson, & Renfrew, 2003	Not a systematic review
Renfrew, McCormick, Wade, Quinn, & Dowswell, 2012	BFI accreditation outcomes not evaluated as a specific subgroup. Review now updated by McFadden et al., 2017
Renfrew, Spiby, D'Souza, Wallace, Dyson, & McCormick, 2007	Not a systematic review of BFI initiative evaluation
Renfrew, Woolridge, & Ross McGill, 2000	Not a systematic review of BFI initiative evaluation
Rollins et al., 2016	Discussion around Sinha et al., 2015
Salera‐Vieira & Zembo, 2016	Looks at history of BFI and implementation, not a systematic evaluation of outcomes after BFI accreditation
Salt & Romano, 2007	Study is about the implementation of the ‘mother‐friendly childbirth initiative’ for which the BFI ten steps are one element of this initiative. Not a SR on the BFI as an intervention and its impact on BR initiation, duration or exclusivity
Schmied, Thomson, Byrom, Burns, Sheehan, & Dykes, 2014	Meta ethnography about setting up BFI, not evaluation of BFI implementation
Sikorski, Renfrew, Pindoria, & Wade, 2003	BFI accreditation outcomes not evaluated as a specific subgroup
Skouteris, Bailey, Nagle, Hauck, Bruce, & Morris, 2017	Not an evaluation of BFI accreditation effectiveness
Spiby, McCormick, Wallace, Renfrew, D'Souza, & Dyson, 2009	Looks at training as an intervention, not BFI accreditation implementation

### REFERENCES

Baby friendly update. Obesity and breastfeeding—A review of the evidence. (2010). The Practising Midwife *,* 13(8), 36–38.20862891

Balogun, O. O., O'Sullivan, E. J., McFadden, A., Ota, E., Gavine, A., Garner, C. D., … MacGillivray, S. (2016). Interventions for promoting the initiation of breastfeeding. Cochrane Database of Systematic Reviews *, Issue* 11, Art. No.: CD001688. 10.1002/14651858.CD001688.pub3 PMC646478827827515

Beake, S., Bick, D., Narracott, C., & Chang, Y. (2017). Interventions for women who have a caesarean birth to increase uptake and duration of breastfeeding: A systematic review. Maternal & Child Nutrition *,* 13, e12390.10.1111/mcn.12390PMC686603527882659

Britton, C., McCormick, F., Renfrew, M., Wade, A., & King, S. (2007). Support for breastfeeding mothers. Cochrane Database of Systematic Reviews, Issue 1, Art. No.: CD001141 10.1002/14651858.CD001141.pub3 17253455

Buccini, G., Pérez‐Escamilla, R., Compte, M. V., Flores, D. (2018) The costs of implementing the Baby‐Friendly Hospital Initiative (BFHI): A systematic review. PROSPERO registration, CRD42018083577 Available from: https://www.crd.york.ac.uk/prospero/display_record.php?ID=CRD42018083577

Carroll, G., Safon, C., Buccini, G., Vilar‐Compte, M., Teruel, G. & Pérez‐Escamilla, R. (2020). A systematic review of costing studies for implementing and scaling‐up breastfeeding interventions: What do we know and what are the gaps? Health Policy and Planning, 35(4) 461–501, DOI: 10.1093/heapol/czaa005 32073628

Cleminson, J., Oddie, S., Renfrew, M. J., & McGuire, W. (2015). Being baby friendly: Evidence‐based breastfeeding support. Archives of Disease in Childhood. Fetal and Neonatal Edition *,* 100(2), F173‐F178. 10.1136/archdischild-2013-304873 25293712

Cramton, R., Zain‐Ul‐Abideen, M., & Whalen, B. (2009). Optimizing successful breastfeeding in the newborn. Current Opinion in Pediatrics *,* 21(3), 386–396. 10.1097/MOP.0b013e32832b325a 19421060

de Oliveira, M. I., Camacho, L. A., & Tedstone, A. E. (2001). Extending breastfeeding duration through primary care: A systematic review of prenatal and postnatal interventions. Journal of Human Lactation *,* 17, 326–343, DOI: 10.1177/089033440101700407 11847902

DeMott, K., Bick, D., Norman, R., Ritchie, G., Turnbull, N., Adams, C., … Taylor, C. (2006). Clinical guidelines and evidence review for postnatal care: Routine postnatal care of recently delivered women and their babies. London: National Collaborating Centre for Primary Care.

Dyson, L., McCormick, F., & Renfrew, M. J. (2005). Interventions for promoting the initiation of breastfeeding. Cochrane Database of Systematic Reviews *, Issue* 2, Article No.: CD001688. DOI: 10.1002/14651858.CD001688.pub2 15846621

Dyson, L., Renfrew, M. J., McFadden, A., McCormick, F., Herbert, G., Thomas, J. (2010). Policy and public health recommendations to promote the initiation and duration of breast‐feeding in developed country settings. Public Health Nutrition *,* 13(1), 137–144. 10.1017/S136898000999067X 19686608

Fallon, A., Crepinsek, M., Hegney, D. & O'Brien, M. (2005). The Baby‐Friendly Hospital Initiative and breastfeeding duration: relating the evidence to the Australian context. Birth Issues, 14(3), 90–95.

Figueredo, S.F., Mattar, M.J.G. & De Vilhena Abrão, A.C.F. (2012). Baby‐friendly Hospital Initiative: A policy of promoting, protecting and supporting breastfeeding. Acta Paulista de Enfermagem, 25(3), 459–463, DOI: 10.1590/S0103-21002012000300022

Forster, D. A., & McLachlan, H. L. (2007). Breastfeeding initiation and birth setting practices: A review of the literature. Journal of Midwifery & Women's Health *,* 52(3), 273–280, DOI: 10.1016/j.jmwh.2006.12.016 17467594

Gli, F. A. A., Spence, D., Lynn, F., Tubman, R., & Sadeq, Z. M. (2019). Baby friendly hospital initiative in the middle east countries: A review of the literature. Indian Journal of Public Health Research and Development *,* 10(10), 902–906. 10.5958/0976-5506.2019.02935.8

Gomez‐Pomar, E., & Blubaugh, R. (2018). The baby friendly hospital initiative and the ten steps for successful breastfeeding. A critical review of the literature. Journal of Perinatology *,* 38(6), 623–632. 10.1038/s41372-018-0068-0 29416115

Guise, J. M., Palda, V., Westhoff, C., Chan, B. K., Helfand, M., Lieu, T. A., & US Preventive Services Task Force. (2003). The effectiveness of primary care‐based interventions to promote breastfeeding: Systematic evidence review and meta‐analysis for the US preventive services task force. Annals of Family Medicine *,* 1, 70–78, DOI: 10.1370/afm.56 15040435PMC1466575

Haroon, S., Das, J. K., Salam, R. A., Imdad, A., & Bhutta, Z. A. (2013). Breastfeeding promotion interventions and breastfeeding practices: A systematic review. BMC Public Health *,* 13(Suppl 3), S20, DOI: 10.1186/1471-2458-13-S3-S20 24564836PMC3847366

Health Canada, the Canadian Paediatric Society, Dietitians of Canada, & Breastfeeding Committee for Canada. (2012). Nutrition for healthy term infants: Recommendations from birth to six months. Canadian Journal of Dietetic Practice and Research: A Publication of Dietitians of Canada = Revue Canadienne De La Pratique Et De La Recherche En Dietetique: Une Publication Des Dietetistes Du Canada *,* 73(4), 204. 10.3148/73.4.2012.204 23217450

Kramer, M. S., Chalmers, B., Hodnett, E. D., Sevkovskaya, Z., Dzikovich, I., Shapiro, S., … Helsing, E. (2001) Promotion of breastfeeding intervention trial (PROBIT): A randomized trial in the Republic of Belarus. JAMA *;* 285(4), 413–420, DOI: 10.1001/jama.285.4.413 11242425

Lubold, A. M. (2017). The effect of family policies and public health initiatives on breastfeeding initiation among 18 high‐income countries: A qualitative comparative analysis research design. International Breastfeeding Journal *,* 12, 1–11. 10.1186/s13006-017-0122-0 28769994PMC5532763

Martens, P. J. (2012). What do Kramer's baby‐friendly hospital initiative PROBIT studies tell us? A review of a decade of research. Journal of Human Lactation: Official Journal of International Lactation Consultant Association *,* 28(3), 335–342. 10.1177/0890334412438264 22584874

McFadden, A., Gavine, A., Renfrew, M. J., Wade, A., Buchanan, P., Taylor, J. L., … MacGillivray, S. (2017). Support for healthy breastfeeding mothers with healthy term babies. Cochrane Database of Systematic Reviews *,* Issue 2, Art. No.: CD001141. 10.1002/14651858.CD001141.pub5 PMC646448528244064

Naylor, A. J. (2001). Baby‐friendly hospital initiative: Protecting, promoting, and supporting breastfeeding in the twenty‐first century. Pediatric Clinics of North America *,* 48(2), 475–483. 10.1016/S0031-3955(08)70039-7 11339166

Patnode, C. D., Henninger, M. L., Senger, C. A., Perdue, L. A., & Whitlock, E. P. (2016). Primary care interventions to support breastfeeding: Updated evidence report and systematic review for the US preventive services task force. JAMA *,* 316(16), 1694–1705, DOI: 10.1001/jama.2016.8882 27784101PMC13475363

Philipp, B. L., & Merewood, A. (2004). The baby‐friendly way: The best breastfeeding start. Pediatric Clinics of North America *,* 51(3), 761–783, DOI: 10.1016/j.pcl.2004.01.007 15157597

Pramono, A., Desborough, J., & Smith, J. (2019). The ten steps to successful breastfeeding policy review. Breastfeeding Review *,* 27(3), 15–28.

Protheroe, L., Dyson, L., & Renfrew, M. J. (2003). The effectiveness of public health interventions to promote the initiation of breastfeeding. London: Health Development Agency.

Renfrew, M. J., McCormick, F. M., Wade, A., Quinn, B., & Dowswell, T. (2012). Support for healthy breastfeeding mothers with healthy term babies. Cochrane Database of Systematic Reviews *,* Issue 5, Art. No.: CD001141. DOI: 10.1002/14651858.CD001141.pub4 PMC396626622592675

Renfrew, M. J., Spiby, H., D'Souza, L., Wallace, L. M., Dyson, L., & McCormick, F. (2007). Rethinking research in breast‐feeding: A critique of the evidence base identified in a systematic review of interventions to promote and support breast‐feeding. Public Health Nutrition *,* 10(7), 726–732, DOI: 10.1017/S1368980007387405 17381919

Renfrew, M. J., Woolridge, M., & Ross McGill, H. (2000). Enabling women to breastfeed: A review of practices which promote or inhibit breastfeeding—With evidence‐based guidance for practice. Norwich: The Stationary Office.

Rollins, N. C., Bhandari, N., Hajeebhoy, N., Horton, S., Lutter, C. K.Martines, J. C., PiwozE. G., RichterL. M.Victora, C.G. (2016). Why invest and what it will take to improve breastfeeding. The Lancet *,* 387, 491–504, DOI: 10.1016/S0140-6736(15)01044-2 26869576

Salera‐Vieira, J., & Zembo, C. T. (2016). Trends in baby‐friendly® care in the united states: Historical influences on contemporary care. The Journal of Perinatal & Neonatal Nursing *,* 30(3), 243–248. 10.1097/JPN.0000000000000176 27465459

Salt, K., & Romano, A. (2007). Step 10: Strives to achieve the WHO/UNICEF ten steps of the baby‐friendly hospital initiative to promote successful breastfeeding. The Coalition for Improving Maternity Services, 16(1), 79S‐80S.10.1624/105812407X173227PMC240913518523679

Schmied, V., Thomson, G., Byrom, A., Burns, E., Sheehan, A., & Dykes, F. (2014). A meta‐ethnographic study of health care staff perceptions of the WHO/UNICEF baby friendly health initiative. Women and Birth *,* 27(4), 242–249. 10.1016/j.wombi.2014.05.005 24957926

Sikorski, J., Renfrew, M. J., Pindoria, S., & Wade, A. (2003). Support for breastfeeding mothers: A systematic review. Paediatric and Perinatal Epidemiology *,* 17, 407–417, DOI: 10.1046/j.1365-3016.2003.00512.x 14629324

Sinha, B., Chowdhury, R., Sankar, M. J., Martines, J., Taneja, S., Mazumder, S., RollinsN., BahlR.Bhandari, N. (2015). Interventions to improve breastfeeding outcomes: A systematic review and meta‐analysis. Acta Paediatrica *,* 104, 114–134. 10.1111/apa.13127 26183031

Skouteris, H., Bailey, C., Nagle, C., Hauck, Y., Bruce, L., & Morris, H. (2017). Interventions designed to promote exclusive breastfeeding in high‐income countries: A systematic review update. Breastfeeding Medicine *,* 12(10), 604–614, DOI: 10.1089/bfm.2017.0065 28885859

Spiby, H., McCormick, F., Wallace, L., Renfrew, M. J., D'Souza, L., & Dyson, L. (2009). A systematic review of education and evidence‐based practice interventions with health professionals and breast feeding counsellors on duration of breast feeding. Midwifery *,* 25(1), 50–61. 10.1016/j.midw.2007.01.006 17418464

## APPENDIX G FULL RESULTS WITHIN EACH INCLUDED SYSTEMATIC REVIEW

**Table:**

Systematic review Total BFI studies (overlap)	Confidence in review findings	Breastfeeding initiation	Duration of exclusive breastfeeding	Duration of any breastfeeding	Health outcomes	Other outcomes
Atchan et al. (2013) 14 (12 in Pérez‐Escamilla et al., 2016, 1 [+9] in Howe‐Heyman & Lutenbacher, 2016, 1 original)	Critically low		Global pre‐post BFHI initiative surveys have shown improved exclusive BF rates in low income countries (study reported in other reviews) 7 cohort studies show BFHI linked to increased exclusivity of BF (outcome from 3× studies not reported in other reviews)	1 large observational study found BFHI associated with increased BF at 1 week (study reported in other reviews) 10 cohort studies show BFHI linked to slightly increased duration of BF, with increased duration with increased number of BFHI steps implemented (outcome from 4× studies not reported in other reviews) Three studies reported breastfeeding rates are influenced by the number of BFHI steps implemented) (1x study not reported in other reviews) No BFHI practices leads to 13 times more likely to cease any BF prior to 6 weeks (study not reported in other reviews)	One RCT shows a range of improved health outcomes, e.g., decreased gastrointestinal infection, but not allergy or asthma. Long term an increase in neuro cognitive development was noted, but no differences in childhood obesity (study reported in other reviews)	
Beake et al. (2012) Beake et al. (2011) 21 + 5 systematic reviews (15 in Pérez‐Escamilla et al., 2016, 5 not BFHI studies/full BFHI studies, 1× original)	Low	7 out of 9 studies found improved initiation rates (outcome of 5 studies not reported in other reviews of which 2 were not full BFHI accreditation studies)	Increased rates of exclusive BF found in hospital in all 4 studies reporting this outcome (outcome of 2 studies not reported within other reviews, but neither were full BFHI accreditation studies) 4 of 4 studies found improved exclusive BF duration (from 1 to 2.5 months longer) (all studies reported in other reviews)	5 out of 6 studies showed improved any BF up to 1 week (although the final one found improved exclusive BF at up to 1 week) (outcome of 2 studies not reported within other reviews, one of which was not full BFHI accreditation study) 3 out of 4 showed increase BF at 2 weeks (outcome of 1 study was not reported within other reviews, but was not a full BFHI accreditation study) 4 studies out of 6 showed higher BF rates up to 1 month PN (outcome of 1 study was not reported within other reviews, but was not a full BFHI accreditation study) 1 study out of 2 showed higher BF rates up to 2 months PN (all studies reported in other reviews) 6 of 7 studies found increased any BF at 3 or 4 months (all studies reported in other reviews) 4 of 5 studies found improved any BF at 6 months (outcome of 1 study was not reported within other reviews, but was not a full BFHI accreditation study)	1 study found decreased gastroenteritis infection and eczema, but no difference in respiratory tract infections (study reported in other reviews)	Maternal BF knowledge was higher in the BFHI group than the control (1× study not in other reviews) Women reported increased BF support in hospital in the intervention group (1× study not in other reviews) Health care professionals' knowledge increased after BFHI training (1x study not in other reviews)
Chung et al. (2008) 2 (2 in Pérez‐Escamilla et al., 2016)	Critically low	No difference in BF initiation with BFHI intervention (study reported in other reviews)	Increased short‐ and long‐term duration of exclusive BF with BFI in 2 studies (studies reported in other reviews)	Increased short and long term duration of any BF with BFI (study reported in other reviews)	Decreased gastrointestinal infection and atopic dermatitis and no difference in respiratory infection in 1 study (study reported in other reviews)	
Fairbank et al. (2000) 2 (1 in Sinha et al., 2015, 1 not full BFI [staff training on the 10 steps])	High	BF initiation increased in one before‐after study (study reported in other reviews)		Increased duration of predominantly BF with BFI up to 4 months in a before after study (study reported in other reviews) No differences noted in before‐after study at 24 months (study reported in other reviews)		Health care provider knowledge of BFI and intention to change practice increased in one RCT (one study not reported in other reviews)
Fallon et al. (2019) 11 (3 in Pérez‐Escamilla et al., 2016, 3× original quantitative, 5× original qualitative)	Low	One cohort study showed women were 10% more likely to initiate BF in units with BFI support than no support (study reported in other reviews)	One cohort study showed women were 28% more likely to exclusively BF at 7 days in units with BFHI accreditation (study reported in other reviews) An intervention study found women were more likely to exclusively BF at 2 weeks and 6 weeks. (study not reported in other reviews, but not a full BFHI accreditation study)	No evidence that BFHI accreditation had an impact on BF prevalence at 1 month (1 cohort study *N* = 18,819) (study reported in other reviews) An intervention study found women were more likely to any BF at 2 weeks, but not at 6 weeks. (study not reported in other reviews, but not a full BFHI accreditation study) Three studies (two intervention and one cross‐sectional observational) provided some evidence that implementation of step 2 (staff training) and step 4 (supporting mothers to initiate and maintain BF) within the **BFCI standards** improves rates of any at 6–8 weeks (2 studies not included in other reviews)		Thematic synthesis of women's experiences (*n* = 214) showed professional support is highly influential in women's experiences of BFI care; BFI may promote unrealistic expectations and not meet women's individual needs; and have an emotional impact especially guilt and feeling pressurised to BF. (5 studies not included in other reviews)
Feltner et al. (2018) 12 (10 in Pérez‐Escamilla et al., 2016, 2 [+1] in Munn et al., 2016)	High	Initiation of any BF 0.5–10% higher in units with BFHI accreditation in 6 cohort studies, but not significant within 4 of these studies (1× study not reported in other reviews) Initiation of exclusive BF 3–56% higher in units with BFHI accreditation in 5 cohort studies (1× study not reported in other reviews)	One RCT showed women had a higher rate of exclusive BF at 3 months (43% vs. 6%) and at 6 months (7.9% vs. 0.6%) (study reported in other reviews) Five cohort studies found benefit of BFHI on exclusive BF at 1 to 2 months (between 4% and 25% increase compared to control) (all studies reported within other reviews)	One RCT showed lower odds of being weaned from BF at 3, 6, 9 and 12 months in BFHI accredited rather than control unit (study reported in other reviews) Eight cohort studies mainly found benefit of BFHI on any BF at 1 to 12 months (outcome from 1× study not reported in other reviews) One cohort found increased odds of weaning before 8 weeks with fewer than 4 BFHI steps than six or more BFHI steps (study reported in other reviews)		
Hannula et al. (2008) 6 (5× in Pérez‐Escamilla et al., 2016, 1× not full BFHI)	Critically low	One RCT showed BFI effective at increasing initiation) (study reported within other reviews)	One USA study showed increased exclusivity with BFI (study reported within other reviews)	1 RCT showed BFI increased BF duration (study reported within other reviews) Three other studies showed longer BF durations with BFHI interventions (all studies reported within other reviews) One study showed women are 8× more likely to discontinue BF with no BFI practices than those experiencing 5 + steps (study reported within other reviews) One study showed no difference between control and intervention but was a low quality study and not full BFHI accreditation intervention		
Howe‐Heyman and Lutenbacher (2016) 25 (16 in Pérez‐Escamilla et al., 2016, 1 (+6) Feltner et al., 2018, 1 (+9) Atchan et al., 2013, 7× original)	Critically low	4 out of 7 studies showed increased initiation. Two of the three studies showing no effect were large cohort studies (outcome from 2× studies not reported in other reviews)	6 out of 13 studies showed increase in short term exclusivity up to 1 month, although exclusivity was poorly defined (outcome from 5× studies not reported in other reviews) 5 out of 10 showed increase in long term exclusivity (outcome from 4× studies not reported in other reviews)	2 out of 8 studies showed increased BF up to 1 month (outcome from 2× studies not reported in other reviews) 9 out of 11 studies showed increase long term BF (over 1 month) (outcome from 3× studies not reported in other reviews)		
Kim et al. (2018) 4 (2 in Pérez‐Escamilla et al., 2016, 1 [+1] in Sinha et al., 2017, 1× original)	Critically low		Exclusive BF up to 6 months OR 5.21 (95% CI 2.15–12.61), 4 studies, *n* = 20,861, *I* ^2^ = 97%) (3 studies reported in another review, 1× study not reported in other reviews but not full BFI study)			
Munn et al. (2016) 16 (5 in Pérez‐Escamilla et al., 2016, 2 (+2) in Sinha et al., 2015, 2 (+1) in Feltner et al., 2018, 6 original, 1 unknown)	Critically low	BFHI increases rates of initiation in 8 studies (outcome from 2× studies not reported in other reviews)	BFHI increases exclusivity of breastfeeding in 8 studies (outcome from 2× studies not reported in other reviews)	BFI practices contributed to increased BF duration, although results should be interpreted with caution due to inconsistencies in documenting BF rates. 5 studies (outcome from 2× studies not reported in other reviews) The higher number of steps implemented appeared to increase the impact on BF outcomes (3× studies not reported in other reviews)	Evidence is limited for the impact of the BFHI on early infant health outcomes (neonatal weight loss, hyperbilirubinemia, hypoglycaemia, and hypothermia). BFHI has been successful in NICU, but impact on late preterm infants less clear (3× studies study not reported in other reviews)	The more training a HCP receives the more likely they are to comply with BFHI practices and philosophy from 4 studies (outcome from 3× studies not reported in other reviews)
Pérez‐Escamilla et al. (2016) 58 (24× original, for overlap see other review results)	Critically low	Quasi experimental evidence (5 out of 5 studies) and observational studies (5 out of 5 studies) suggests higher BF initiation rates with BFHI accreditation (outcome from 2× studies not reported in other reviews)	Most RCT evidence (2 studies), quasi experimental (11 out of 12 studies) and observational studies (16 out of 17) show improved exclusive BF with BFHI intervention when evaluated at up to 6 months. (outcome from 16× studies not reported in other reviews) RCT evidence (3 studies) and some low quality quasi‐experimental evidence suggests good implementation of step 10 (home peer support) is necessary for BF improvements (outcome from 3x studies not reported in other reviews)	Most RCT (2 out of 3 studies), quasi experimental (11 out of 12 studies) and observational studies (15 out of 16) show improved any BF with BFHI intervention when evaluated at up different time points up to 12 months (outcome from 12× studies not reported in other reviews) Some evidence that the more BFHI steps implemented the longer the impact on BF rates (outcome from 3x studies not reported in other reviews)	Evidence from 1 RCT: Decreased gastroenteritis and atopic eczema at 1 year of age, but no significant difference for respiratory tract infection, infant weight outcomes or head circumference at 12 months. Follow up to 6.5 years—positive impact on children's IQ and academic performance, but not dental health, child behaviour, blood pressure, anthropometric measures, allergy or asthma risk. Observational evidence: Women reported experiencing mastitis less when delivering in a baby friendly hospital (study not reported in other reviews) Women more likely to remain amenorrhoeic at 6 months postpartum after BFHI implementation (study not reported in other reviews)	
Renfrew et al. (2010) Renfrew et al. (2009) 4 (1× in Sinha et al., 2015, 3× original (1 of which was organisation of care [not full BFI] and another not all aspects of BFI)	Low		Exclusive BF at 2 weeks RR 4.20 (95% CI 1.53–11.50, 1 study, *n* = 84, *I* ^2^ N/A) (study reported in other reviews) Exclusive BF at hospital discharge (in neonates admitted to NICU) RR 1.52 (95% CI 1.24–1.86, 1 study, *n* = 495, *I* ^2^ N/A) (outcome from study not reported in other reviews) Two further low quality studies found no difference in exclusive breastfeeding at hospital discharge (outcome from 2x studies not reported in other reviews, however 1 was organisation of care [not BFI] the other was not all aspects of BFI)	Any breastmilk during first week of enteral feeds RR 2.15 (95% CI 1.63–2.84, 1 study, *n* = 227, *I* ^2^ N/A) (study reported in other reviews) Any BF at 2 weeks RR 2.36 (95% CI 1.39–4.00, 1 study, *n* = 84, *I* ^2^ N/A) (study reported in other reviews) Two further low quality studies found no difference in any breastfeeding at hospital discharge (both studies not reported in other reviews, but 1 was organisation of care [not BFI] the other was not all aspects of BFI)		
Sinha et al. (2017) 10 (7 in Sinha et al., 2015, 1 (+1) in Kim et al., 2018, 2× original, 1 of which was not full BFI implementation)	Critically low	Initiation within 1 hour of delivery OR 1.45 (1.12, 1.89), 7 studies, *n* = NR, *I* ^2^ = 75%) (3× estimates from 2 × studies not reported in other reviews)	Exclusive breastfeeding up to 1 month OR 0.84 (95% CI 0.35–2.03, 1 study, *n* = NR, *I* ^2^ = N/A) (study reported in other reviews) Exclusive breastfeeding at 1–5 month OR 2.89 (95% CI 1.73–4.80, 8 studies, *n* = NR, *I* ^2^ = 98%) (outcome from 1× study not reported in other reviews)	Continued breastfeeding (from 6 to 23 months) OR 1.69 (95% CI 1.28–2.24, 3 studies, *n* = NR, *I* ^2^ = 55%) (all studies reported in other reviews)		
Sinha et al. (2015) 24 (16× in Pérez‐Escamilla et al., 2016, 2 (+2) in Munn et al., 2016, 2 (+5) in Sinha et al., 2017, 1 (+9) In Beake et al., 2011, 3× original)	Critically low	Initiation within 1 h of delivery higher with BFHI than control RR 1.20 (95% CI 1.11–1.28, 10 studies, *n* = NR, *I* ^2^ = 79%) (outcome from 2× studies not reported in other reviews)	BFHI support increased exclusive breastfeeding (any time point in the first 6 months) compared to control RR 1.49 (95% CI 1.33–1.68, 15 estimates from 13 studies, *n* = NR, *I* ^2^ = 97%) (outcome from all studies reported within other reviews)	BFHI support had the most impact out of any interventions on any BF up to 6 months compared to control RR 1.66 (95% CI 1.34–2.07, 13 studies, *n* = NR, *I* ^2^ = 98%) (outcome from 3× studies not reported in other reviews) BFHI support had no significant effect on continued BF from 6 to 23 months RR 1.26 (95% CI 0.96–1.64, 3 studies, n = NR, I^2^ = 46%) (outcome from 2x studies not reported in other reviews)		

*Note*: Shaded reviews or outcomes are duplicated results from the same study/studies within other reviews.

Abbreviations: BF, breastfeeding; BFHI, Baby Friendly Hospital Initiative; BFI, Baby Friendly Initiative; CI, 95% confidence interval; IQ, Intelligence Quotient; N/a, not applicable; NICU, Neonatal intensive Care Unit; NR, not reported; OR, odds ratio; RCT, Randomised Controlled Trial; RR, relative risk; *I* ^2^, measure of heterogeneity between included studies; *n*, number of included studies.
